# Garden-based interventions and early childhood health: a protocol for an umbrella review

**DOI:** 10.1186/s13643-019-1229-8

**Published:** 2019-12-06

**Authors:** Kara Skelton, Ann Herbert, Sara E. Benjamin-Neelon

**Affiliations:** 1 Johns Hopkins Bloomberg School of Public Health, 624 N. Broadway, HH904, Baltimore, MD 21205 USA; 2 Johns Hopkins Bloomberg School of Public Health, 624 N. Broadway, HH755, Baltimore, MD 21205 USA

**Keywords:** Gardening, Young children, Farm to preschool

## Abstract

**Background:**

Garden-based interventions have the potential to impact young children’s health in a number of ways, including enhancing dietary intake, increasing outdoor physical activity, diversifying the gut microbiome, and promoting general wellbeing. A number of recent systematic reviews have either included or focused on garden-based interventions for young children. However, most prior reviews including young children only focus on one health outcome or one setting, making a full summary of prior research assessing the impact of garden-based interventions nonexistent. As such, this umbrella systematic review aims to synthesize the literature on health outcomes of garden-based interventions for young children.

**Methods:**

This protocol outlines the systematic steps we will take to conduct an umbrella review on health-related outcomes of garden-based interventions in children younger than 6 years of age. We will systematically search PubMed, PsycINFO, ERIC, CINAHL, Embase, Scopus, OVID-Agricola, and CAB Direct, including all systematic reviews and meta-analyses fitting the pre-determined inclusion/exclusion criteria. We will double screen at each phase of the review: title/abstract, full text, data extraction, and quality appraisal. We will assess the quality of included reviews using A Measurement Tool to Assess Systematic Reviews (AMSTAR 2). Based on the potential for stark variability in what how reviews report child health outcomes, we will analyze the reviews both narratively and quantitatively, reporting summary of findings tables and iteratively mapping the results. This protocol aligns with the Preferred Reporting Items for Systematic Reviews and Meta-Analyses Protocols statement (PRISMA-P).

**Discussion:**

This umbrella review aims to summarize the role that garden-based interventions play in health promotion for young children. We will focus on a number of diverse child health outcomes in an effort to comprehensively synthesize the evidence to inform future garden-based interventions, research, and policy.

**Systematic review registration:**

PROSPERO CRD42019106848

## Background

Garden-based interventions have the potential to improve a wide range of child health outcomes, including enhancing dietary intake, increasing outdoor physical activity, diversifying the gut microbiome, and promoting general wellbeing. This may be due, in part, to the potential of garden-based interventions to promote healthy eating and physical activity, while enriching children with food origin and food systems knowledge [1–3]. Research on garden-based interventions in youth and adult populations have shown associations between gardening and reductions in anger, stress, anxiety, and body mass index (BMI). They have also demonstrated improvements in cognitive functioning, life satisfaction, mood, and overall quality of life [4–7]. There is growing evidence that garden-based interventions may have additional benefits for young children, such as stress reduction and improved mental health and academic performance, suggesting interventions which are garden-based may be able to improve multiple aspects of health simultaneously [5, 8]. However, previous systematic reviews have primarily focused on single health outcomes [9–12], leaving large gaps in what is known about the holistic health and wellbeing impacts of gardening programs for young children.

Prior garden-based intervention studies in young children have found some improvement in dietary outcomes, such as fruit or vegetable intake, willingness to try new foods, and even BMI [13–15]. Younger children may be more willing to taste and accept novel foods than older children [16], and exposure to fruits and vegetables by age 5 years is vital for establishing habitual consumption later in life [17]. Previous research has also shown that hands-on experiences through garden-based interventions may increase fruit and vegetable consumption more than an intervention that merely increases availability [3, 18]. Additionally, garden-based interventions have been utilized as a form of therapy for an array of disorders and diseases, such as autism spectrum disorder [19] and childhood cancer [20]. The benefits of improved dietary and physical activity behaviors and increased nutrition knowledge acquired from these interventions may have the potential to seep through into other child health outcomes, such as enhanced academic performance and improved mental health [10, 21]. However, few review articles have considered and assessed diverse child health outcomes within the same article

While a number of systematic reviews on garden-based interventions for children exist [5, 6, 8, 10, 11, 22–24], there has yet to be a comprehensive umbrella review that summarizes the wide array of health benefits of garden-based interventions for young children. As noted above, existing systematic reviews on garden-based interventions for children focus primarily on single health outcomes, such as fruit and vegetable intake [11] or academic performance [10]. Additionally, most reviews examined only one type of gardening program (e.g., farm-to-preschool) rather than exploring the multiple settings in which garden-based interventions can be implemented [24]. Although there is a published review that looks more broadly at the health impacts of gardening in school-aged children, it included only one study with preschool-aged children [8]. Additionally, due to the diversity in settings, types, and benefits of garden-based interventions, we aim to holistically evaluate and summarize existing systematic reviews on garden-based interventions and health outcomes for young children in a single umbrella review.

The following question will guide this umbrella review: What role can garden-based interventions play in health promotion for children aged 6 years and younger? To successfully answer this question, we will explore the following objectives:

1. To identify and synthesize existing review articles, ranging from narrative reviews to meta-analyses, on garden-based interventions for young children;
2. To determine which garden-based interventions are effective at improving child health and well-being outcomes;
3. To identify the most prominent measures used to detect and assess the health and wellbeing impacts of garden-based interventions in young children;
4. To critically evaluate available garden-based interventions on child health outcomes both narratively and quantitatively;
5. To identify gaps in the literature and to highlight potential areas of improvement for the scientific field of garden-based interventions, including, but not limited to study design, measurement, and child health outcomes.

## Methods

### Protocol development

This umbrella review protocol follows the Joanna Briggs Institute Methodology for Umbrella Reviews [25]. This protocol was also developed to align with the Preferred Reporting Items for Systematic Reviews and Meta-Analyses Protocols (PRISMA-P) 2015 statement [26] (Additional file 1) and has been registered with the PROSPERO database for systematic reviews (#CRD42019106848).

### Review methodology

Given the existence of systematic reviews on garden-based interventions that focus on or include young children, we will conduct an umbrella review, in accordance with the Joanna Briggs Institute Methodology for Umbrella Reviews. The systematic review methodology outlined in this paper will be used to strategically locate, synthesize, and evaluate published systematic review- and meta-analysis-level evidence on the role of garden-based interventions in the health promotion of young children. Umbrella reviews are able to systematically assess the highest levels of evidence for an overall topic, while evaluating the quality of the evidence concurrently [25, 27]. Therefore, this umbrella review highlights strengths, as well as gaps, in the evidence for garden-based interventions.

We will assess the quality of included systematic reviews on garden-based interventions, including both randomized studies and non-randomized studies, and with and without a control or comparison group, appraising methodological characteristics through the use of A Measurement Tool to Assess Systematic Reviews (AMSTAR 2). Through this critical appraisal tool, this umbrella review will also enable researchers and other stakeholders to determine the quality of existing systematic review-level evidence on garden-based interventions for young children.

### Inclusion criteria

We used the population, intervention, context, outcome and study design (PICOS) structure in formulating the scope of this umbrella review [27]. This enabled us to precisely delineate a priori inclusion criteria for the umbrella review. We will apply the inclusion criteria at both the review and individual study level. For example, there may be a review that meets our inclusion criteria, but further examination at the individual study level reveals there are no studies included within the review that meet inclusion criteria. In this case, we would exclude the review. In other words, there must be at least one individual-level study that meets all inclusion criteria for us to include the review.

#### Participants

This umbrella review will include systematic reviews that include children younger than 6 years of age. For inclusion, reviews do not have to be focused solely on our age range of interest. However, children younger than 6 years must be within the included age range of at least one included article included in the review. We will not exclude participants based on gender or any other socioeconomic-related factors.

#### Intervention

This umbrella review will include systematic reviews that focus on or include garden-based interventions. As there is no single definition of garden-based interventions due to their complexity and variation in type and setting, we have defined this term for the purposes of this review. Thus, we define garden-based interventions as any intervention that engages children in active learning about nutrition, food systems, agriculture, or environmental health through connections with outside fruit or vegetable gardens or farms, raised garden beds, greenhouses, container gardens, microfarms, or other alternative gardening methods. We will also include farm-to-school and farm-to-child care programs, which typically link children with fruit and vegetables from local farms or gardens. For young children, garden-based interventions can occur in an array of settings, including homes, early care and education programs (e.g., center-based child care or preschool), community centers, community gardens, afterschool programs, and summer camps. Similarly, there are numerous ways in which garden-based interventions can be implemented. Some garden-based interventions are standalone programs, and others may be integrated into broader nutrition education programs that incorporate additional interventions simultaneously. For the purposes of this umbrella review, we will include reviews of any garden-based interventions that meet the above criteria. This criterion is intentionally broad to allow for a range of reviews and interventions to be included in this review.

#### Context

We will not employ any limitations related to the context in which the garden-based interventions take place. Reviews can include garden-based interventions that take place in any county or climate, or in a rural or urban setting. Similarly, the garden-based interventions could be designed for any type of young child, including those of any gender and socioeconomic status. Garden-based interventions that are focused on children with certain health conditions, special developmental and or psychological considerations, will also be included.

#### Outcomes

We will consider reviews that include any child-level health or wellbeing outcomes. These include but are not limited to weight or BMI, the gut microbiome, health-related behaviors (e.g., diet, physical activity, social interaction), academic (e.g., knowledge or cognition), and mental health. We will also include qualitative outcomes, including major themes and concepts relating to garden-based interventions, where reported. We will also consider adverse or unintended consequences where noted in reviews. We will include reviews that report both child and parent-level health outcomes, but only the child-level outcomes will be extracted and included in the analysis. We will exclude reviews that focus solely on parent-, school-, or community-related outcomes.

#### Types of studies

For this review, we will include only systematic reviews. Included reviews could have conducted meta-analysis or narrative synthesis as part of their analysis. We will define a systematic review with guidance from the PRISMA-P 2015 statement. A systematic review will be defined as a review which (a) has an explicit set of aims; (b) employs a reproducible methodology, including a systematic search strategy and selection of studies; and (c) is a systematic presentation and synthesis of the characteristics and findings of included studies [26]. We will exclude review articles that do not meet this definition of a systematic review. We will also exclude individual primary studies. There are no limitations for study designs included in the reviews; any systematic review reporting data about child-level health and overall wellbeing benefits of garden-based interventions for children younger than 6 years will be included. We will include systematic reviews that are randomized (e.g., randomized controlled trial), quasi-randomized, and non-randomized designs (e.g., pre-post design, non-randomized trial). We will exclude systematic reviews that examine qualitative studies. We will include only full review articles published after 1990, as is best practice with umbrella review methodology [27]. Additionally, we will include only peer-reviewed literature (i.e., we will exclude dissertations and conference abstracts) (Table 1).

**Table 1:** Study inclusion and exclusion criteria

	Inclusion criteria	Exclusion criteria
Study type	Systematic reviews, with or without meta-analysis	All other study types (e.g., qualitative systematic review, non-systematic reviews, individual studies)
Study period	Reviews published in or after 1990	Reviews published prior to 1990
Participants	Children younger than 6 years of age	Children 6 years of age and older
Primary outcomes	Any child-level health or wellbeing outcome	Any non-child-level outcomes (e.g., parent or community-related outcomes)
Intervention/program type	Garden-based interventions or programs	Reviews that do not include garden-based interventions or programs

### Search strategy

#### Database search

We will search the following databases from 1990 onward: PubMed, PsycINFO, ERIC, CINAHL, Embase, Scopus, OVID-Agricola, and CAB Direct. Additionally, we will search systematic review databases, including the Cochrane Register of Systematic Reviews, the Joanna Briggs Institute Database of Systematic Reviews and Implementation Reports, and PROSPERO. We will also search the first 200 results of Google Scholar, when sorted in relevance ranking, for review and meta-analyses articles. Additionally, we will conduct reference list and citation searches for all included articles. Similarly, when included systematic reviews have been cited more than 200 times, the citation searches will be limited to the first 200 most recent citations. As Cochrane reviews are updated frequently and may be published in peer-reviewed journals in addition to the Cochrane database, we may retrieve more than one review published by the same author(s) on the same topic (e.g., an update of an existing review). If this circumstance arises, we will only include the most recent version of the review. However, we will cite to previous versions of the review.

#### Search terms

We will search the abovementioned electronic databases using database-specific controlled vocabulary and key terms. We developed this search strategy using terms for gardening and young children that have been utilized in previous reviews [8, 23, 24, 28] as well as additional terms that were selected to capture the breadth of the body of literature. To ensure completeness, we drafted the search strategy in collaboration with a Medical Librarian who specializes in systematic reviews and an expert in early childhood gardening research.

### Pilot search

We conducted a pilot search strategy for all databases listed above (Table 2). We utilized key terms and controlled vocabulary for each database. We used five key review papers as “targets” to ensure the pilot search located the types of articles we wanted to include. The pilot search included all search terms. As we wanted to make sure we were not losing a large number of systematic reviews due to the date restrictions, we ran the pilot search without date restrictions. As we did not find any relevant systematic reviews on this topic for any database prior to 1990, we will include the restriction in the final search strategy. Additionally, we slightly adapted search terminology to ensure we capture all relevant farm-to-school and farm-to-childcare reviews. Adaptation involved the inclusion of additional terminology and removal of terminology that was not relevant to yield all potentially eligible reviews. The final search strategy to be used for the umbrella review is presented in Additional file 2.

**Table 2:** Pilot search results, from 1990 to January 9, 2019

	PubMed	PsycINFO	ERIC	CINAHL	Scopus	Embase	Google Scholar	CAB Direct	Agricola
Search strategy details in Additional file 2	10, plus MeSH terms	10, plus DE terms	10, plus SU terms	10, plus MJ terms	10	10, plus Emtree terms	1 AND 2 AND 3	10, plus PG terms	1 AND 2 AND 3
Number of hits	12,601	518	610	498	1,753	92	200	378	0
Target papers									
Savoie-Roskos	**✓**	**✓**		**✓**	**✓**	**✓**			
Davis	**✓**					**✓**			
Ohly		**✓**		**✓**	**✓**		**✓**		
Masset					**✓**			**✓**	

### Study screening

The lead and second reviewer will carry out the initial database search. To manage the blinded title and abstract screening process, we will utilize Covidence Software (Covidence Systematic Review Software, Veritas Health Innovation, Melbourne, Australia). As citations are imported into Covidence, they will automatically be de-duplicated based on an exact match of the title, date, and author. Following the comprehensive search, screening of the titles and abstracts will occur independently by three reviewers split into two teams. Within Covidence, each citation will be screened, using a priori inclusion and exclusion criteria and then categorized as “No,” “Maybe” or “Yes.” Through Covidence, the citation will be automatically filtered into one of three lists: “Irrelevant,” “Resolve Conflicts,” and “Full text review.” For a citation to be added to the “Irrelevant” list, a “No” must be cast by both reviewers; if there is a disagreement between the two reviewers, the citation will move to the “Resolve Conflicts” list. A citation will move automatically to “Full text review” with any combination of “Maybe” and “Yes” received by a team of reviewers. Disagreements between reviewers will be resolved using consensus, and by a third reviewer if necessary.

Once the list of citations moving forward to full-text review is complete, the research team will gather articles in their full-text, PDF form with assistance from a medical librarian. During full text screening, both reviewers must agree on a final inclusion/exclusion decision as well as the accompanying rationale. If the authors cannot determine eligibility after full-text review, we will contact the review authors to assist in determining eligibility. We will document all reasons for exclusion throughout the full text screening phase in Covidence. After exhausting all efforts to retrieve full-text for a citation, the full text cannot be retrieved, it will be excluded. Additionally, should there be duplicates at this level, we will exclude them. Only those articles meeting all inclusion criteria will move forward for data abstraction. The number of included studies from search through data extraction will be automatically created using a PRISMA flow diagram within Covidence.

### Data extraction

During the full text data extraction phase, two reviewers will independently extract article data directly into Covidence. Using recommendations for relevant data fields for umbrella reviews from Aromataris et al. 2015 [27], we will collect the following data, at minimum, from each eligible systematic review: (1) citation details; (2) purpose/objectives of the included review; (3) review methodology (e.g., meta-analysis, narrative synthesis); (4) study population (e.g., age, demographic characteristics), setting (e.g., country, setting of garden-based intervention), and context; (5) search strategy and results (e.g., number of databases sources and searched, date range, inclusion of gray literature); (6) number of included studies, citation, type, and country of origin of studies; (7) child-level health and wellbeing outcomes reported that are relevant to the umbrella review questions; (8) funding sources for each review; (9) main findings relevant to the review question; and (10) comments or notes regarding included studies. For each review article, we will also extract information for individual primary studies meeting inclusion criteria, including, but not limited to citation details, child characteristics, setting, intervention type and design, results, limitations, and conclusions to enable us to account for overlap at the primary study level. We will be limited to data extraction at the individual study level based on what is presented in the review. The data extraction form is included as Additional file 3. We will contact corresponding authors for any missing data or for clarification of unclear items.

### Quality appraisal

In addition to data extraction, a quality appraisal for each systematic review will be conducted using the AMSTAR 2, which is updated to allow for both randomized and observational studies [29]. The recent update to the original AMSTAR tool addresses the more modern need for policy and research decision making that account for “real-world observational evidence” [29]. AMSTAR 2 is an appraisal tool consisting of 16 items with the following response options: Yes, Partial Yes, No. The AMSTAR has been evaluated and shown to be both valid and reliable [30]. Please note that AMSTAR 2 was not intended to be scored, and as such we will not score this tool. To evaluate quality, two reviewers will independently extract relevant data based on the AMSTAR 2 for each included article. Any disagreements between reviewers will be resolved among themselves first through discussion and by a third reviewer if the reviewers are unable to achieve consensus. We will not exclude reviews based on results of their quality assessment. Rather, we will conduct the quality assessment to critique the strength of evidence generated.

## Confidence in evidence

For included studies, we will report on confidence in findings using the information presented in each review. This could be in the form of a quality of evidence tool, such as the Grading of Recommendations Assessment, Development, and Evaluation (GRADE) measures [31]. However, there are many additional tools that existing reviews can use to assess the confidence in evidence, which may cause variations in how this aspect is reported in reviews. As such, we will report on the exact tool used for included reviews and their associated findings, iteratively making the decision on how to display confidence in findings based on the information reported in each individual review. If a review is included in which the authors did not conduct an assessment of confidence in evidence, the review team will not conduct any de novo assessments. In this instance, there will be no confidence in evidence presented for that review.

## Reporting of findings

For this umbrella review, we will report a summary of findings from all included reviews based on data synthesis, presenting a comprehensive overview of what is known in the literature on the role of garden-based interventions in health promotion for children aged 6 years and younger. We will create the summary of findings tables from extracted data, directly mapping findings to our research questions per the Joanna Briggs Institute methodology for conducting an umbrella review [25] and the Cochrane Handbook’s Methodology for conducting an overview of reviews [32]. We will take a mixed-methods approach to synthesizing the review literature, utilizing both qualitative methods (e.g., narrative synthesis) and quantitative methods (e.g., numerical patterns or associations). In line with the research questions guiding this umbrella review, we plan to highlight the strengths and weakness of included systematic reviews, as well as describe any evidence gaps we identify. We also plan to report on effectiveness of garden-based interventions. For this, we will present a grid of intervention components and child health and well-being outcomes, noting directionality of each outcome. We plan to summarize the setting, outcome measures, numbers of children, and pooled results from each review, including implications for future research and practice through tables and narration, as appropriate. When reporting findings in tabular form, we will present child health outcomes across included reviews, stratifying tables by review methodology (e.g., all evidence gained from reviews of randomized controlled trials) and child health outcome (e.g., reporting evidence on academic performance separately from nutrition outcomes). For child health outcomes, we anticipate reporting tabular results in the following categories: child nutrition outcomes, academic performance, education, mental health and social skills, gut microbiome, and physical activity. For child nutrition outcomes, we anticipate a need to break down results further, in which case we will report results in the following categories: (1) intake, reporting separately on fruit and vegetable intake as allowed; (2) selection, reporting fruit and/or vegetable selection and preference outcomes; and (3) biometric and anthropometric outcomes (e.g., body mass index, wasting, etc.) Where tabular presentations of results are presented, they will be accompanied by detailed descriptions. As we are including a broad range of health outcomes, we expect included reviews to report on child health outcomes in different ways and will be limited to what is reported. We will aim to report health outcomes stratified by the following age groups: infancy (less than 12 months of age), toddlers (12 months of age–less than 36 months of age), and preschool (3 years of age–less than 6 years of age). However, we will be limited in this stratification by how included reviews report on health outcomes by age. For example, if a review stratifies findings by age (e.g., preschool, school-age), we will report our results accordingly. Similarly, if a review does not separate results by age, we will only be able to report what is reported in the review. In this instance, results that cannot be disaggregated by age will be reported separately within our review.

Additionally, we will create a table summarizing the AMSTAR 2 results for each included review. As is best practice, we will report the entire umbrella review in accordance with PRISMA guidelines.

Due to the potential for overlap of primary studies in the review articles, we will report the number of times individual studies are included across multiple reviews. We will systematically identify any occurrences of overlap across systematic reviews by noting individual studies included in more than one review. We will also explore the consistency of reporting at the individual level across reviews [33] to determine if there is any overlap. We will explore this through the use of The Cochrane Handbook’s template for mapping individual primary studies contained within included systematic reviews [32]. For example, one review article may report on one specific child health outcome of an individual study, while another review includes the same study but reports on a different (perhaps secondary) health outcome. In this case, we would not consider this overlap. However, if reviews are reporting the same outcomes from the same study, we will highlight this overlap. If we do find there is overlap, we will calculate the corrected covered area [34] and report on this measure in the review paper. We will also consider overlap when interpreting results of the review.

## Discussion

For this umbrella review, we aim to (1) identify and synthesize existing review and meta-analysis articles on garden-based interventions for young children; (2) identify the most prominent measures used to detect and assess the impacts of garden-based interventions in young children; (3) critically evaluate the available evidence both narratively and quantitatively; and (4) identify gaps in the literature and describe areas of improvement for the scientific field of garden-based interventions, including, but not limited to study design, measurement, and child health outcomes. In doing so, we will provide a very comprehensive overview of what is known in the literature about the health and wellbeing impacts of gardening programs for young children.

This umbrella review has several strengths. First, this umbrella review fills a considerable gap in the literature by providing a holistic overview of existing evidence of the health and wellbeing benefits of garden-based interventions for young children, identifying strengths of current evidence and highlighting areas for improvement. To date, this type of umbrella review does not exist. Second, this review will examine knowledge gaps in the field and elaborate on how these gaps could be addressed by future research. Summarizing this information will be an asset to both researchers and public health professionals aiming to improve the health and wellbeing of young children. Additionally, this umbrella review will be conducted using the most systematic procedures available at this time. Adhering to these guidelines helps ensure that we procedure a high-quality umbrella review that will be a useful and trusted resource for interested parties.

Anticipated challenges for this review include the need to extract relevant information from existing review articles. We realize there are numerous benefits to garden-based interventions throughout the life span. However, we only focused on children younger than 6 years, as this is an area where garden-based interventions in an ECE, community, or home setting could make a substantial impact. We limited our search to peer-reviewed literature published after 1990, but do not believe this will exclude any relevant studies based on the pilot search strategy. However, we realize the exclusion of non-peer reviewed literature may eliminate high-quality reviews, which is another limitation. We will elaborate on additional limitations in regard to findings in the narration of the review. Another limitation of this review will be the potential for study overlap across reviews. Knowing this potential risk, we will examine and report on any overlap in the review.

Despite anticipated limitations, conducting this umbrella review on the role of gardening for health promotion in young children could be of great importance for researchers, public health professionals, and policymakers. By summarizing the current evidence for and simultaneously identifying strengths, weaknesses, and limitations for garden-based interventions, we hope to strengthen the quality of future research in this area. Further, we hope to support and highlight evidence-based interventions that improve the health and wellbeing of young children.

## Supplementary information

**Additional file 1.** PRISMA-P Checklist.

**Additional file 2.** Search Strategy.

**Additional file 3.** Data Extraction Form.

## Data Availability

N/A

## Abbreviations

AMSTAR: A Measurement Tool to Assess Systematic Reviews
ECE: Early care and education
PICO: Population, intervention, context, outcome and study design
RoB: Risk of bias
