# Supplementary material for: Garden-based interventions and early childhood health: a protocol for an umbrella review
Source: Syst Rev. 2019 Dec 6;8:310. doi: 10.1186/s13643-019-1229-8 (PMC6896344; doi:10.1186/s13643-019-1229-8)
Supplement: Supplementary file 3 — Additional file 3. Data Extraction Form. [file 13643_2019_1229_MOESM3_ESM.docx]

**Additional File 3. Data Extraction Form**

**STUDY DETAILS**

SPONSORSHIP SOURCE

COUNTRY

SETTING

COMMENTS

**AUTHOR CONTACT DETAILS**

NAME

INSTITUTION

EMAIL

ADDRESS

**ADDITIONAL DATA**

CONFLICT OF INTEREST

JOURNAL

PUBLISHED DATE

**METHODS**

DESIGN

GROUP

ASSESSED STUDY QUALITY

CONCLUSIONS BY AUTHORS FOR REVIEW

DATABASES SEARCHED

DATE RESTRICTIONS FOR SEARCH

FUTURE RESEARCH

INCLUDED GRAY LITERATURE

KEYWORDS USED FOR SEARCH

META-ANALYSIS PERFORMED

NUMBER OF INCLUDED ARTICLES

NUMBER OF REVIEWERS

OUTCOMES OF INTEREST

OVERALL TOPIC AREA OF INTEREST (E.G. OBESITY, NUTRITION, GARDENING, ETC.)

PROSPERO/COCHRANE REGISTRATION NUMBER

PURPOSE/AIMS AND RESEARCH QUESTIONS

**POPULATION**

INCLUSION CRITERIA

EXCLUSION CRITERIA

GROUP DIFFERENCES

AGES INCLUDED IN REVIEW

COUNTRIES INCLUDED

HEALTH OUTCOMES INCLUDED

INTERVENTION TYPE INCLUDED

LANGUAGES INCLUDED

SETTING(S) INCLUDED

SUBPOPULATIONS INCLUDED IN REVIEW

YEARS INCLUDED

**INDIVIDUAL STUDY DATA**

SPONSORSHIP SOURCE

Methods

COUNTRY

SETTING

Population

Intervention Characteristics

Results

Limitations

Conclusions

COMMENTS

**ADDITIONAL DATA**

CONFLICT OF INTEREST

JOURNAL

PUBLISHED DATE
